# Disparities in program enrollment and employment outcomes for veterans with psychiatric and co-occurring substance use disorders referred or enrolled for VHA vocational rehabilitation

**DOI:** 10.3389/fpsyt.2023.1200450

**Published:** 2023-07-13

**Authors:** Matthew E. Sprong, Heaven Hollender, Yu-Sheng Lee, Lee Ann Rawlins Williams, Zach Sneed, Amir Garakani, Frank D. Buono

**Affiliations:** ^1^Edward Hines Jr. VA Medical Center, Hines, IL, United States; ^2^School of Public Management and Policy, University of Illinois Springfield, Springfield, IL, United States; ^3^Department of Health Sciences, Indiana University-Purdue University Indianapolis, Indianapolis, IN, United States; ^4^School of Integrated Sciences, Sustainability, and Public Health, University of Illinois Springfield, Springfield, IL, United States; ^5^Rehabilitation and Human Services, College of Education and Human Development, University of North Dakota, Grand Forks, ND, United States; ^6^School of Health Professions, Texas Tech University Health Sciences Center, Lubbock, TX, United States; ^7^Department of Psychiatry and Behavioral Health, Greenwich Hospital, Greenwich, CT, United States; ^8^Department of Psychiatry, School of Medicine, Yale University, New Haven, CT, United States

**Keywords:** employment, substance use disorders, veterans, vocational rehabilitation, mental health, co-occurring disorders, psychiatric disorders, Department of Veteran Affairs

## Abstract

**Introduction:**

The purpose of the study was to investigate factors that influence vocational rehabilitation program enrollment and employment at discharge of veterans with psychiatric and co-occurring alcohol and other substance use disorders enrolled at a veteran health administration (VHA) medical center.

**Methods:**

A sample of 2,550 veteran patients referred for VHA vocational rehabilitation between 2016 and 2021 were examined for the current study. The current study was classified as quality improvement/assurance, thus resulting in exempt research by the U.S. Department of Veteran Affairs Institutional Review Board.

**Results:**

Veterans with active alcohol use disorders (AUDs) and co-occurring depression, anxiety, post-traumatic stress disorder, or bipolar disorders were less likely to be enrolled for vocational rehabilitation program services compared to those without these co-occurring diagnoses. Veterans with AUD (active & in-remission status combined into one category) and a diagnosis of anxiety were less likely to be employed at discharge compared to veterans with AUDs and no anxiety diagnosis (anxiety diagnosis – 3.5% vs. no anxiety diagnosis – 5.8%).

**Discussion:**

VHA vocational rehabilitation can be an effective intervention to assist veterans in reintegrating back into the community. Yet, there appears to be some disparities in the program enrollment and employment at discharge, depending on the nature of the psychiatric diagnosis. Investigating the factors contributing (mediating or moderating) to these discrepancies are needed. Although it appears access is not the issue in being referred for vocational rehabilitation services, other factors are likely contributing to program entry.

## Introduction

The trajectory of substance use disorders (SUDs) is expected to rise in the United States (U.S.), and recent research examining 30 geographically-representative VA healthcare systems (6,000 Veteran patients) demonstrated alcohol as the most commonly reported substance (24% use in past 12 months, 11% daily use in past 3 months, with 10% meeting DSM-5 criteria for alcohol use disorder), followed by cannabis (42% lifetime use, 12% use in past 12 months, 5% daily use in past 3 months, 3% met DSM-5 criteria for cannabis use disorder) (1). With significant increases in mortality rates and high prevalence of substance use disorders (SUDs), the administration of President Joseph Biden and Vice-President Kamala Harris have taken action to allocate federal dollars in support of the prevention and reduction of substance abuse disorders (SUDs) (2). Such funding has: (a) allowed for the development of evidence-based interventions; and (b) enhanced service-delivery models by providing funding for additional peer supports and treatment expansion. An estimated 2.3 percent (481,000) of veterans are diagnosed with a mental illness and SUD (3). The literature demonstrates an increased risk of medical hospitalizations when these diagnoses co-occur (4). Evidence has shown that other issues emerging from co-occurring diagnoses, such as homelessness, unemployment, social exclusion, low self-efficacy in community reintegration and military-to-civilian transition, and self-esteem, which all impact quality of life (5).

### Employment issues: psychiatric and substance use disorders

Members of our armed forces put their lives on hold to protect and serve the U.S. Therefore, it is essential to provide mental health services to reduce the challenges associated with transitioning or return into civilian life. Prevalence rates have shown that 1 in 10 veterans are diagnosed with SUDs (6) and approximately 91% have gone without physical or psychological treatment in 2020 (3). Of the 1.1 million veterans living with a SUD, 25% struggle with illicit drug use and 80% struggle with alcohol use disorders (AUDs) (7). The Substance Abuse Mental Health Services Administration (SAMHSA) has indicated that the most common mental disorders seen in SUD treatment include anxiety and mood disorders, schizophrenia, bipolar disorder, major depressive disorder, conduct disorders, post-traumatic stress disorders, and attention deficit hyperactivity disorders (8). Current estimates show that 1 in 4 adults living with serious mental problems also has an issue with substance use (9). For veterans, mental health (MH) and co-occurring SUD diagnosis rates have been at 5.7% or 1.1 million (10).

Research has shown that in 2020, approximately 5.2 million veterans experienced a behavioral health condition and more than half with a psychiatric disorder did not receive treatment in the prior year (11). As previously mentioned, there are several factors that can impact independent living, quality of life, and having a sense of belonging when veterans have psychiatric diagnoses or SUDs (12, 13). Research has conclusively shown that employment is one factor that is lower for veterans with these diagnoses and combination of diagnoses, with these veterans having lower labor force participation (14–16). Within the Veteran Health Administration (VHA), all medical centers are required to have a vocational rehabilitation (VR) [referred to as Compensated Work Therapy] to improve vocational functioning and employment outcomes of Veterans (17).

### VHA vocational rehabilitation

VR is a method of assisting veterans to obtain the skills and tools needed to successfully secure and maintain competitive employment. A primary focus is on removing employment-related barriers for veterans that have difficulty obtaining or maintaining employment (18, 19). VHA VR programs are expected to adhere to VHA Policy Directive 1163.02, which was released on August 19, 2019. Prior research has shown that implementation of this policy did not impact program enrollment rates for veterans with SUDs, but veterans with AUDs were less likely to be enrolled prior to the implementation of VHA Policy Directive 1163 (18). This policy explicitly states the following: “no discriminatory practices are acceptable for Veterans with SUD or AUDs “regardless of duration of sobriety, routine vocational testing, or required time in a treatment program or clinical services prior to participation in VHA Vocational Rehabilitation” (p. 43). Additionally, under VHA Policy Directive 1163, all VHA medical facilities must offer two specialty programs within VHA VR including supported employment (SE) and transitional work (TW) (17).

SE is a program that serves two groupings of veterans, including: (1) veterans with serious mental illness or medical conditions (e.g., active psychosis, schizoaffective disorder, severe traumatic brain injury, blindness, spinal cord injury); and (2) veterans with significant employment issues as it relates to SUDs (e.g., attendance issues) are eligible for the SE program. Veterans enrolled in TW participate with work restoration services in actual work settings. The goal is to provide vocational supports and resources needed for successful transition to competitive employment. TW is for veterans that do not have a serious mental illness or medical condition as aforementioned but have significant employment related impairments. The Community Based Employment Services (CBES) program is optional for each VHA VR program, and accessible when made readily available. Veterans with mental health conditions or physical disabilities who have a history of sporadic employment, difficulty maintaining a job, or initiating and following through on their job search, or who require a range of supports to obtain or maintain competitive work, are eligible. However, veterans enrolled in these programs do not have the diagnoses as required to be eligible for SE, and have the knowledge, skills, and abilities to engage in competitive employment without the need of work restoration services (TW).

VR is a crucial component of the comprehensive care provided to veterans with and without disabilities, and eligibility for VHA VR includes (a) medically cleared to work and participate in the program, and (b) goal of obtaining competitive employment (17). The efficacy of including VR as a part of treatment has been well-supported in the successful recovery of veterans with SUDs (20–22). Through the provision of employment-related services, such as vocational evaluation, career counseling, and job training, VR helps veterans regain their independence and achieve meaningful employment. Research has shown that VR can significantly improve the employment outcomes of veterans. Bond and colleagues (23) noted veterans who participated in a VR program had a higher likelihood of finding employment and greater job satisfaction than those who did not participate in VR.

In addition to improving employment outcomes, VR can also enhance the mental health and well-being of veterans. By providing a sense of purpose and accomplishment, employment can help veterans to rebuild their self-esteem and self-confidence, and to reduce symptoms of depression and anxiety. Furthermore, vocational rehabilitation can help veterans to develop social connections and establish a sense of belonging within their communities. As reflected upon by the U.S. (17), employment is a critical component of a Veteran’s overall wellness and recovery, and VR plays an important key role in supporting successful reintegration into civilian life. Overall, vocational rehabilitation is a vital component of the care provided to veterans with psychiatric and/or medical disabilities. By improving employment outcomes and enhancing mental health and well-being, VR supports veterans to regain their independence and achieve meaningful lives.

### Vocational rehabilitation and mental health

The negative association between employment and psychiatric conditions and SUDs is well-documented, and research has shown that employment participation decreases as the symptoms of the conditions increase as psychiatric and SUD conditions can serve as a significant barrier. For example, after examining nearly 100,000 veterans, Zivin et al. (24) found that veterans with bipolar disorder, depression, post-traumatic stress disorder (PTSD), schizophrenia, or SUDs were more likely to be unemployed, have a disability or be retired than employed. Other studies have shown that veterans with SUD diagnoses or who were homeless at program entry were more likely to be employed at discharge, while receipt of public support income and severe mental illness decreased the inclusion in the competitive labor market (22, 25–28).

National databases connected with VHA VR have been examined to evaluate its efficacy in employment and healthcare outcomes, and research findings have shown improvement in employment obtainment and retention (22) and lower healthcare needs (e.g., fewer visits in VHA vocational rehabilitation, outpatient mental health, homelessness program, and medical hospitalizations) post-discharge (25). However, the co-occurring/co-morbid nature of having both a psychiatric and SUD diagnosis concurrently lowers participation in competitive employment (29). Other non-veteran related research has shown that employment is a strong predictor of SUD treatment compliance and continued abstinence post-discharge (30).

Despite the evidence showing how psychiatric and SUD/AUD diagnoses can impact a veteran’s independent functioning and psychological well-being, the purpose of this study was to focus on the impact of having these diagnoses as they co-occur in terms of how it effects VHA vocational rehabilitation program enrollment and employment at discharge. Studies have shown that employment at discharge decreases when a veteran presents a SUD/AUD diagnosis or a mental health condition (18, 19), but there is limited research examining the impact when both co-occur for veterans enrolled in VR within the VA healthcare system.

## Materials and methods

### Data source and procedures

The Institutional Review Board (IRB) for the U.S. Department of Veteran Affairs indicated that this was a retrospective and quality assurance study, and therefore no approval (exempt) was needed if no identifying information was released for publication. The co-author’s university granted additional IRB endorsement (approval #13460) as exempt status to verify that institutional support was provided for the current study. The current study included data from a VHA VR program located within VISN-12 and accounted for veterans referred for services between 2016 and 2020. All veteran information was recorded by VR counselors in an access database that was used for programmatic evaluation (e.g., gender, race, homelessness status). It is possible that this data were entered incorrectly (data input errors), and to verify the data’s accuracy, the primary author used the U.S. Department of Veteran Affairs’ Computerized Patient Record System (CPRS) and Joint Legacy Viewer (JLV) – includes data from medical care at other VA medical facilities across the country if applicable, and a search through CPRS progress notes and consults. The database used during the intake interview did not account for the durational period of last SUD/AUD usage (active, early remission, sustained remission) nor psychiatric diagnoses per the DSM 5 TR. Therefore, information was collected from CPRS and JLV to properly code the diagnoses (e.g., early remission from AUD or SUD is 3–12 months post last use). Lab testing was used to verify last usage per the urine drug screening criteria.

#### Predictor and outcome variables and data analysis

The DSM 5-TR classifies SUD into three categories (active usage, early remission, sustained remission) to provide details on the severity of the disorder (31). Researchers examining employment challenges for Veterans have generally combined all drug and alcohol related diagnoses into one category (29, 32), without differentiating whether the diagnoses are in remission or an active problem (18, 19). In the current study, it was intended to (1) evaluate program enrollment rates and employment rates at discharge by only differentiating between SUD or AUD and no SUD or AUD, and (2) separating SUD and AUD into none, active usage, early remission, and sustained remission. Due to low frequencies within early remission and sustained remission, the variable was condensed into no usage, active usage, and inactive usage (sustained and early remission SUD and AUD). Prior literature has shown that veterans with psychiatric disorders experience significant employment-related barriers (22). Therefore, psychiatric disorders was used as the other predictor variable. Additionally, two outcome variables were utilized within the current study, including program enrollment and employment status at discharge. For program enrollment, this variable was dichotomized into enrolled and not enrolled.

All veterans that had received a consult for VHA vocational rehabilitation were included in the data analysis. Oftentimes, veterans are not enrolled for services for several reasons such as (1) job obtainment prior to program start, (2) no longer wishing to work competitively, (3) having medical issues arise resulting in a cancellation until medical complications are no longer problematic, or (4) no longer interested in receiving services and seeking competitive employment. All veterans in the database were coded as 0 = not enrolled and 1 = enrolled. For veterans enrolled in the program and discharged, the coding was dichotomized: 0 = discharged without employment, 1 = discharged with employment. The outcome variables of VHA vocational rehabilitation program enrollment and employment at discharge, were evaluated by using a binary logistic regression analysis. In addition, the interaction between SUD and psychiatric disorder was utilized to examine the impact for veterans with both diagnoses on program enrollment and employment at discharge.

## Results

The sample consisted of 2,550 veteran patients referred to VHA VR between 2016 and 2021. Of these, the majority of the sample were biologically male (*n* = 1,740, 87.92%), and had a mean age of 55.24 years (SD 13.16 years). Most of the participants had a high school diploma (*n* = 2,231, 87.49%), and were Black or African American (*n* = 1,083, 46.28%), followed by white non-Hispanic (*n* = 991, 42.35%), Hispanic or Latino (*n* = 189, 8.08%), and Asian or Other Pacific Islander (*n* = 25, 1.07%). When considering the presence of clinical diagnosis, Major Depressive Disorder [F33 code, recurrent] (*n* = 1,019, 40.0%) and PTSD [F43.10] (*n* = 763, 29.9%) had the highest frequencies, followed by Generalized Anxiety Disorder [F41.1] (*n* = 391, 15.3%), and adjustment disorder [F43.20] (*n* = 220, 8.6%). A total of 607 (27.96%) veterans reported that they were on the supplemental nutrition assistance program (SNAP), while 63 veterans (2.54%) were noted as being enrolled in the homeless program and were classified as homeless at program consult. Table 1 provides a breakdown of demographic information. The data are categorized SUD/AUD (none, active, inactive) and representative of psychiatric disorders (i.e., adjustment, anxiety, depression, PTSD) with the highest frequencies.

**TABLE 1:** Frequency of psychiatric conditions and co-occurring SUD/AUD disorders and other supplemental information.

	Total	Adjustment *n* (%)		Anxiety *n* (%)		Depression *n* (%)		PTSD *n* (%)	
		**No**	**Yes**	**No**	**Yes**	**No**	**Yes**	**No**	**Yes**
	2,550	2,330 (91.4)	220 (8.6)	2,159 (84.7)	391 (15.3)	1,531 (60.0)	1,019 (40.0)	1,787 (70.1)	763 (29.9)
**SUD/AUD**									
No	1,370	1,257 (53.9)	113 (51.4)	1,176 (54.5)	194 (49.6)	862 (56.3)	508 (49.8)	996 (55.7)	374 (49.0)
Yes	1,180	1,073 (46.1)	107 (48.6)	983 (45.5)	197 (50.4)	669 (43.7)	511 (50.2)	791 (44.3)	389 (51.0)
**SUD/AUD**									
None	1,370	1,257 (53.9)	113 (51.4)	1,176 (54.5)	194 (49.6)	862 (56.3)	508 (49.8)	996 (55.7)	374 (49.0)
Inactive	401	364 (15.6)	37 (16.8)	340 (15.7)	61 (15.6)	235 (15.3)	166 (16.3)	276 (15.5)	125 (16.4)
Active	779	709 (30.4)	70 (31.8)	643 (29.8)	136 (34.8)	434 (28.4)	345 (33.9)	515 (28.8)	264 (34.6)
Subtotal SUD of psychiatric condition (SUD & AUD combined)		107 (4.2)		197 (7.7)		511 (20.0)		389 (15.3)	
		**SUD**	**AUD**	**SUD**	**AUD**	**SUD**	**AUD**	**SUD**	**AUD**
***N***		43 (40.2)	64 (59.8)	60 (30.5)	137 (69.5)	164 (32.1)	347 (67.9)	109 (28.0)	280 (72.0)
**Age (mean, SD)**		54.0 (14.2)	52.5 (12.4)	54.7 (14.6)	53.0 (13.9)	57.0 (12.5)	55.2 (12.9)	55.7 (13.2)	53.6 (13.2)
**Biological sex**									
Male		38 (88.4)	63 (98.4)	47 (78.3)	132 (96.4)	139 (84.8)	328 (94.5)	94 (86.2)	260 (92.9)
Female		5 (11.6)	1 (1.6)	13 (21.7)	5 (3.6)	25 (15.8)	19 (5.5)	15 (13.8)	20 (7.1)
**Year of education**									
<12 years		1 (2.3)	1 (1.6)	1 (1.7)	6 (4.4)	8 (4.9)	16 (4.6)	5 (4.6)	12 (4.3)
12–15 years		33 (76.7)	47 (73.4)	39 (65.0)	101 (73.7)	124 (75.6)	238 (68.6)	83 (76.1)	202 (72.1)
≥16 years		9 (21.0)	16 (25.0)	20 (33.3)	30 (21.9)	32 (19.5)	93 (26.8)	21 (19.3)	66 (23.6)
**Food stamps**									
No		36 (83.7)	46 (74.2)	51 (85.0)	111 (84.1)	125 (76.2)	248 (73.4)	92 (84.4)	220 (81.2)
Yes		7 (16.3)	16 (25.8)	9 (15.0)	21 (15.9)	39 (23.8)	90 (26.6)	17 (15.6)	51 (18.8)
**Service period**									
Korean		0 (0.0)	0 (0.0)	0 (0.0)	0 (0.0)	0 (0.0)	0 (0.0)	0 (0.0)	1 (0.4)
OEF/OIE		11 (26.2)	22 (35.5)	25 (41.7)	64 (49.2)	48 (29.8)	125 (37.8)	50 (45.8)	160 (58.8)
Persian Gulf/Gulf War		10 (23.8)	11 (17.7)	17 (28.3)	25 (19.2)	40 (24.9)	66 (19.9)	19 (17.4)	43 (15.8)
Post-Vietnam		11 (26.2)	18 (29.1)	10 (16.7)	26 (20.0)	44 (27.3)	94 (28.4)	20 (18.4)	36 (13.2)
Vietnam Era		10 (23.8)	11 (17.7)	8 (13.3)	15 (11.6)	29 (18.0)	46 (13.9)	20 (18.4)	32 (11.8)
**Branch of military**									
Airforce		3 (7.0)	9 (14.1)	5 (8.3)	12 (8.8)	22 (13.4)	38 (11.0)	11 (10.1)	21 (7.6)
Army		17 (39.5)	32 (50.0)	25 (41.7)	67 (48.9)	79 (48.2)	168 (48.4)	62 (56.9)	162 (58.3)
Coast Guard		1 (2.3)	0 (0.0)	0 (0.0)	1 (0.7)	0 (0.0)	2 (0.6)	0 (0.0)	2 (0.7)
Marine Corps		7 (16.3)	13 (20.3)	8 (13.3)	25 (18.3)	28 (17.1)	69 (20.0)	17 (15.6)	48 (17.3)
Navy		15 (34.9)	10 (15.6)	22 (36.7)	32 (23.4)	35 (21.3)	70 (20.2)	19 (17.4)	45 (16.2)
**Service-connected disability**									
No service connection		29 (67.4)	33 (54.1)	29 (49.2)	69 (51.5)	79 (49.7)	171 (50.7)	31 (29.0)	87 (31.3)
Service connection		14 (32.6)	28 (45.9)	30 (50.8)	65 (48.5)	80 (50.3)	166 (49.3)	76 (71.0)	191 (68.7)
**Driver’s license**									
No		9 (20.9)	12 (18.8)	12 (20.0)	25 (18.7)	42 (25.6)	78 (22.7)	18 (16.6)	64 (23.1)
Yes		34 (79.1)	52 (81.2)	48 (80.0)	109 (81.3)	122 (74.4)	265 (77.3)	90 (83.4)	213 (76.9)
**Racial categories**									
American Indian-Alaskan Native		2 (4.8)	0 (0.0)	1 (1.8)	1 (0.8)	2 (1.3)	1 (0.3)	1 (0.9)	0 (0.0)
Asian		2 (4.8)	0 (0.0)	1 (1.8)	0 (0.0)	1 (0.6)	3 (0.9)	2 (1.9)	2 (0.8)
Black non-Hispanic		14 (33.3)	29 (46.8)	24 (42.9)	44 (33.6)	81 (52.6)	132 (40.1)	51 (48.6)	104 (39.1)
White Hispanic		5 (11.9)	6 (9.7)	2 (3.6)	12 (9.2)	10 (6.5)	27 (8.2)	11 (10.5)	24 (9.0)
Native Hawaiian/Pacific Islander		0 (0.0)	0 (0.0)	0 (0.0)	0 (0.0)	1 (0.6)	0 (0.0)	1 (0.9)	1 (0.4)
White non-Hispanic		18 (42.9)	27 (43.5)	27 (48.2)	72 (55.0)	56 (36.4)	163 (49.5)	38 (36.2)	133 (50.0)
Other		1 (2.4)	0 (0.0)	1 (1.8)	2 (1.5)	3 (2.0)	3 (0.9)	1 (0.9)	2 (0.8)
**Employment status**									
No		13 (44.8)	24 (49.0)	22 (51.2)	53 (68.0)	59 (54.1)	114 (55.6)	40 (54.8)	97 (59.2)
Yes		16 (55.2)	25 (51.0)	21 (48.8)	25 (32.0)	50 (45.9)	91 (44.4)	33 (45.2)	67 (40.8)

### Program enrollment

Program enrollment was analyzed using a chi-square test to compare the impact of SUDs (active, inactive, none), AUDs (active, inactive, none), psychiatric diagnoses (diagnosis, no diagnosis: adjustment, anxiety, depression, PTSD, Bipolar, schizophrenia, schizoaffective), and the co-occurrence of these diagnoses. As shown in Table 2, depression, anxiety, adjustment disorder, and PTSD were significant less likely to enroll in vocational rehabilitation when the veteran also had a SUD diagnosis (note: active, early remission, sustained remission were one category to be consistent with prior research). When splitting SUD and AUD diagnoses into 3 levels (none, active, remission), findings showed that veterans with active AUD combined with a diagnosis of depression or anxiety or adjustment disorder or PTSD or Bipolar disorder were significantly less likely to be employed at discharge. Veterans with an active SUD diagnosis and co-occurring psychiatric diagnosis had similar employment rates at discharge compared to veterans without these diagnoses.

**TABLE 2:** Chi-squared test for program enrollment by SUDs, AUDs, and psychiatric conditions.

	Program enrollment		*p*-value
	**Enrolled**	**Not enrolled**	
SUDs			0.7288
No	1,226 (70.3)	571 (70.9)	
Yes	519 (29.7)	234 (29.1)	
AUDs			0.8748
No	1,202 (68.9)	552 (68.6)	
Yes	543 (31.1)	253 (31.4)	
SUDs			0.6904
None	1,226 (70.2)	571 (70.9)	
Inactive	130 (7.5)	65 (8.1)	
Active	389 (22.3)	169 (21.0)	
AUDs			0.2740
None	1,202 (68.9)	552 (68.6)	
Inactive	183 (10.5)	100 (12.4)	
Active	360 (20.6)	153 (19.0)	
Depression			0.0057^$^
No	1,015 (58.2)	515 (64.0)	
Yes	729 (41.8)	290 (36.0)	
Anxiety			0.1400
No	1,464 (83.9)	694 (86.2)	
Yes	280 (16.1)	111 (13.8)	
Adjustment disorder			0.0031^$^
No	1,574 (90.3)	755 (93.8)	
Yes	170 (9.7)	50 (6.2)	
PTSD			0.0202^$^
No	1,197 (68.6)	589 (73.2)	
Yes	547 (31.4)	216 (26.8)	
Bipolar			0.7613
No	1,605 (92.0)	738 (91.7)	
Yes	139 (8.0)	67 (8.3)	
Schizophrenia			0.6361
No	1,726 (99.0)	795 (98.8)	
Yes	18 (1.0)	10 (1.2)	
Schizoaffective			0.8997
No	1,717 (98.5)	792 (98.4)	
Yes	27 (1.5)	13 (1.6)	
SUD combined with			
Depression	216 (12.4)	104 (12.9)	0.7015
Anxiety	91 (5.2)	43 (5.3)	0.8940
Adjustment disorder	55 (3.2)	17 (2.1)	0.1406
PTSD	164 (9.4)	72 (8.9)	0.7130
Bipolar	41 (2.4)	24 (3.0)	0.3468
Schizophrenia	5 (0.3)	7 (0.9)	0.0456^$^
Schizoaffective	3 (0.2)	4 (0.5)	0.2170
AUD combined with			
Depression	212 (12.2)	135 (16.8)	0.0016^$^
Anxiety	81 (4.6)	56 (7.0)	0.0160^$^
Adjustment disorder	53 (3.0)	11 (1.4)	0.0122^$^
PTSD	176 (10.1)	104 (12.9)	0.0334^$^
Bipolar	45 (2.6)	32 (4.0)	0.0555
Schizophrenia	5 (0.3)	4 (0.5)	0.4758
Schizoaffective	9 (0.5)	6 (0.8)	0.5780
Active AUD combined with			
Depression	133 (7.6)	90 (11.2)	0.0031^$^
Anxiety	52 (3.0)	41 (5.1)	0.0081^$^
Adjustment disorder	37 (2.1)	6 (0.8)	0.0122^$^
PTSD	110 (6.3)	75 (9.3)	0.0064^$^
Bipolar	24 (1.4)	23 (2.9)	0.0097^$^
Schizophrenia	5 (0.3)	3 (0.4)	0.7131
Schizoaffective	7 (0.4)	5 (0.6)	0.5349
Active SUD combined with			
Depression	166 (9.5)	85 (10.6)	0.4098
Anxiety	69 (4.0)	35 (4.4)	0.6404
Adjustment disorder	40 (2.3)	13 (1.6)	0.2651
PTSD	120 (6.9)	60 (7.5)	0.5972
Bipolar	28 (1.6)	19 (2.4)	0.1873
Schizophrenia	5 (0.3)	5 (0.6)	0.3039
Schizoaffective	3 (0.2)	4 (0.5)	0.2170

^$^Statistically significant.

### Psychiatric disabilities and co-occurring alcohol and other substance use disorders

The alcohol and other substance use disorders diagnosis was split into active and inactive (in remission) to examine the influence (active, inactive, none) on program enrollment for veterans who additionally have co-occurring psychiatric diagnoses. A chi-square test was performed (see Table 2) and findings revealed that veterans with active AUD combined with depression (7.6 vs. 11.2%, *p* = 0.0031), anxiety (3.0 vs. 5.1%, *p* = 0.0081), PTSD (6.3 vs. 9.3%, *p* = 0.0064), and bipolar disorder (1.4 vs. 2.9%, *p* = 0.0097) were less likely to be enrolled for program services compared to veterans without these diagnoses. Veterans with adjustment disorder (2.1 vs. 0.8%, *p* = 0.0122) were more likely to be enrolled for program services.

A logistic regression analysis (see Table 3) comparing the outcome variable of enrollment (i.e., enrolled, not enrolled) indicated veterans with active AUD were less likely (OR = 0.67, 95% CI = [0.52–0.87], *p* = 0.0021) to be enrolled for program services compared to veterans without AUD. When including the interaction terms in the regression model (see Table 4), it was observed that the program enrollment was different in active AUD veterans with and without depression (*p* < 0.0001), anxiety (*p* = 0.0006), PTSD (*p* = 0.0011), and bipolar disorder (*p* = 0.0002). Specifically, active AUD veterans with depression (OR = 0.40, 95% CI = [0.26–0.63]), anxiety (OR = 0.53, 95% CI = [0.31–0.88]), PTSD (OR = 0.52, 95% CI = [0.36–0.80]), and bipolar disorder (OR = 0.21, 95% CI = [0.10–0.43]) were less likely to be enrolled for program services than those without these diagnoses.

**TABLE 3:** Logistic regression of association of program enrollment, active/inactive AUD or SUD, and psychiatric conditions.

	Odds ratio	95% confidence interval	*p*-value
Age	1.01	0.99–1.02	0.1564
Gender (Male vs. Female)	0.80	0.56–1.13	0.1983
**Race/Ethnicity**			
White vs. others	1.05	0.74–1.48	0.8436
Black-Non-Hispanic vs. others	1.04	0.74–1.48	0.8729
Education years	1.02	0.97–1.07	0.4802
Active AUD*	0.67	0.52–0.87	0.0021^$^
Inactive AUD*	1.05	0.81–1.34	0.7317
Active SUD*	1.20	0.86–1.66	0.2884
Inactive SUD*	1.28	0.89–1.84	0.1757
Depression*	0.95	0.77–1.19	0.6798
Anxiety*	1.15	0.86–1.54	0.3562
Adjustment disorder*	1.14	0.78–1.66	0.5068
PTSD*	0.96	0.77–1.21	0.7260
Bipolar*	0.75	0.53–1.07	0.1156
Schizophrenia*	0.68	0.28–1.66	0.3997
Schizoaffective*	0.64	0.29–1.42	0.2696

*No is the reference group.

^$^Statistically significant.

**TABLE 4:** Logistic regression of association of program enrollment and active AUD of interaction effect with psychiatric conditions.

	Estimate	Standard error	Odds ratio	95% confidence interval	*p*-value
Age	0.0044	0.00438	1.00	0.99–1.01	0.3146
Gender (Male vs. Female)	−0.0883	0.089	0.92	0.77–1.09	0.3214
**Race/Ethnicity**					
White vs. others	0.0527	0.0819	1.05	0.90–1.24	0.5198
Black-Non-Hispanic vs. others	−0.0119	0.0822	0.99	0.84–1.16	0.8848
Education years	0.0164	0.027	1.02	0.96–1.07	0.5429
Active AUD*	−0.7528	0.1229	0.47	0.37–0.60	<0.0001^$^
Depression*	−0.1675	0.0659	0.85	0.74–0.96	0.0110^$^
Anxiety*	−0.0388	0.0829	0.96	0.82–1.13	0.6398
Adjustment disorder*	0.0842	0.0972	1.09	0.90–1.32	0.3863
PTSD*	−0.1155	0.066	0.89	0.78–1.01	0.0804
Bipolar*	−0.3804	0.1087	0.68	0.55–0.85	0.0005^$^
Schizophrenia*	−0.1774	0.2318	0.84	0.53–1.32	0.4440
Schizoaffective*	−0.2677	0.2085	0.77	0.51–1.15	0.1991
Active AUD × Depression	−0.2868	0.0651	0.40^#^	0.26–0.63	<0.0001^$^
Active AUD × Anxiety	−0.2837	0.0824	0.53^#^	0.31–0.88	0.0006^$^
Active AUD × PTSD	−0.2138	0.0655	0.52^#^	0.36–0.80	0.0011^$^
Active AUD × Bipolar	−0.4048	0.1084	0.21^#^	0.10–0.43	0.0002^$^

*No is the reference group.

^$^Statistically significant.

^#^Odds ratio shown in the table was estimated based on active AUD as well as with psychiatric diagnosis vs. without diagnosis.

### Employment status at program discharge

A chi-square test of independence showed that veterans with a history of SUDs (see Table 5) had no difference in employment rates at discharge compared to veterans with no SUD diagnosis (30.9% with no SUD diagnosis vs. 29.1% with SUD diagnosis, *p* = 0.4441). The same findings occurred when comparing veterans with (31.2%) and without (31.9%) an AUD diagnosis and employment rates at discharge. When only examining psychiatric diagnoses, veterans without an adjustment disorder had higher rates of employment at discharge, but no other psychiatric diagnosis comparisons yielded significant differences. The interaction between psychiatric diagnosis and SUD or AUD yielded significant findings for employment rates at discharge for veterans diagnosed with bipolar disorder and SUDs, where the SUD category was categorized into no diagnosis, SUD diagnosis. When examining veterans with and without AUD and psychiatric diagnosis, veterans with anxiety and AUD (no diagnosis, AUD diagnosis) had significant differences. SUD and AUD diagnosis were then split into three levels, including no diagnosis, active diagnosis, and in-remission diagnosis. A logistic regression analysis comparing the outcome variable of employment (i.e., employment at discharge, no employment at discharge) demonstrated veterans with bipolar disorder were more likely (OR = 1.55, 95% CI = [1.01–2.40], *p* = 0.0492) to be employed at discharge compared to veterans without this disorder (see Table 6).

**TABLE 5:** Chi-squared test for employment at discharge by SUDs, AUDs, and psychiatric conditions.

	Employment at discharge		*p*-value
	**No**	**Yes**	
SUDs			0.4441
No	647 (70.9%)	490 (69.1%)	
Yes	266 (29.1%)	219 (30.9%)	
AUDs			0.7765
No	628 (68.8%)	483 (68.1%)	
Yes	285 (31.2%)	226 (31.9%)	
Depression			0.8982
No	536 (58.7%)	414 (58.4%)	
Yes	377 (41.3%)	295 (41.6%)	
Anxiety			0.6606
No	764 (83.7%)	599 (84.5%)	
Yes	149 (16.3%)	110 (15.5%)	
Adjustment disorder			0.0117^$^
No	839 (91.9%)	625 (88.2%)	
Yes	74 (9.10%)	84 (11.8%)	
PTSD			0.4314
No	626 (68.6%)	499 (70.4%)	
Yes	287 (31.4%)	210 (29.6%)	
Bipolar			0.0099^$^
No	824 (90.2%)	655 (93.8%)	
Yes	89 (9.80%)	44 (6.20%)	
Schizophrenia			0.3721
No	901 (98.7%)	703 (99.2%)	
Yes	12 (1.30%)	6 (0.80%)	
Schizoaffective			0.0071^$^
No	893 (97.8%)	705 (99.4%)	
Yes	20 (2.20%)	4 (0.60%)	
SUD combined with			
Depression	117 (12.8%)	89 (12.6%)	0.8751
Anxiety	51 (5.60%)	34 (4.80%)	0.4785
Adjustment disorder	24 (2.60%)	26 (3.70%)	0.2301
PTSD	83 (9.10%)	68 (9.60%)	0.7310
Bipolar	28 (3.10%)	11 (1.60%)	0.0481^$^
Schizophrenia	3 (0.30%)	2 (0.30%)	1.0000
Schizoaffective	2 (0.20%)	1 (0.10%)	1.0000
AUD combined with			
Depression	114 (12.5%)	91 (12.8%)	0.8340
Anxiety	53 (5.80%)	25 (3.50%)	0.0333^$^
Adjustment disorder	24 (2.60%)	25 (3.50%)	0.2949
PTSD	97 (10.6%)	67 (9.50%)	0.4364
Bipolar	31 (3.40%)	13 (1.80%)	0.0548
Schizophrenia	4 (0.40%)	1 (0.10%)	0.3939
Schizoaffective	7 (0.80%)	2 (0.30%)	0.3137

^$^Statistically significant.

**TABLE 6:** Logistic regression of association of employment at discharge and psychiatric conditions.

	Odds ratio	95% confidence interval	*p*-value
Age	1.00	0.99-1.01	0.6016
Gender (Male vs. Female)	1.32	0.92-1.87	0.1289
**Race/Ethnicity**			
White vs. others	1.38	0.95-2.01	0.3720
Black-Non-Hispanic vs. others	1.51	1.04-2.20	0.0543
Education years	0.88	0.83-0.93	< 0.0001^$^
Active AUD*	0.84	0.63-1.12	0.2239
Inactive AUD*	0.88	0.68-1.15	0.3583
Active SUD*	1.13	0.79-1.62	0.4899
Inactive SUD*	1.14	0.76-1.72	0.5300
Depression*	1.01	0.80-1.28	0.9330
Anxiety*	1.10	0.81-1.49	0.5618
Adjustment disorder*	0.72	0.49-1.05	0.0907
PTSD*	1.10	0.86-1.40	0.4709
Bipolar*	1.55	1.01-2.40	0.0492^$^
Schizophrenia*	1.35	0.46-3.96	0.5842
Schizoaffective*	2.74	0.74-10.1	0.1308

*No is the reference group. ^$^Statistically significant.

## Discussion

The benefits of VHA vocational rehabilitation, including the recovery from psychiatric and SUD diagnoses, is well-documented. Additional research reflects improved independent functioning and lowering of the need for medical, psychological, vocational, and homeless program services through the VHA (22, 25, 26, 28). The current study results yielded program enrollment disparities for veterans with a history of AUD diagnoses and a history of depression, adjustment disorder, and PTSD. Although research has shown the effectiveness that VR can have on independent functioning for veterans (20, 22), the intervention cannot be effective for the veterans that are not enrolled, thus indicating a need for research to (a) identify access and treatment enrollment issues, and (b) an intervention to eliminate these identify issues. The co-occurring diagnoses examined in the current study showed that the combination of psychiatric disorders with the presence of a AUD diagnosis resulted in a lower chance of being enrolled for VHA VR (compared to veterans without these diagnoses).

### Implications for clinical practice and psychological research

VR is a vital component of the care provided to veterans with or without medical and psychiatric disabilities. By improving employment outcomes and enhancing mental health and well-being, VR supports veterans to regain their independence and achieve meaningful lives. As previously noted, veterans who participated in a VR program had a higher likelihood of finding employment and greater job satisfaction than those who did not participate in VR. In addition to improving employment outcomes, VR can also enhance the mental health and well-being of veterans with a disability by providing a sense of purpose and accomplishment, rebuild their self-esteem and self-confidence, and to reduce symptoms of depression and anxiety.

Some potential contributions of this study to the field of rehabilitation could include improved understanding of the unique challenges faced by veterans with co-occurring diagnoses when attempting to enroll in vocational rehabilitation programs and obtain employment. By identifying the specific obstacles that these individuals face, researchers and practitioners can develop more targeted interventions and support services to help them succeed. In addition, the study suggests the need for increased awareness for integrated treatment approaches that address both mental health and physical health issues simultaneously. The findings of this study could highlight the importance of providing comprehensive, coordinated care for veterans with complex medical histories. Finally, implications suggest the need for enhanced ability to measure the effectiveness of vocational rehabilitation programs for veterans with co-occurring diagnoses. By examining the impact of these programs on employment outcomes, researchers can determine which interventions are most effective and identify areas where further improvements are needed. Overall, the study has the potential to inform the development of more effective interventions and support services for veterans with co-occurring diagnoses, ultimately improving their employment prospects and quality of life.

As mentioned, previous research necessitates a focus on challenges endured when veterans are referred for vocational services and why program enrollment rates compared to referrals for VHA VR services (e.g., scheduling of intake interview and treatment plan session). Research must (1) identify reasons why Veteran do not enroll in VHA VR after a referral is made, (2) impact of program engagement on VHA VR enrollment, and (3) the efficacy of providing concurrent vocational rehabilitation services while a Veteran is enrolled in mental health and/or substance use counseling services, rather than having a focus placed on vocational needs toward the end of their treatment regimen (note: not all treatment is post-MH and/or SUD counseling services, but further investigation is needed). State-federal (public) VR programs that are designed for civilians have shown higher SUD and or MH treatment compliance when employment is obtained, higher abstinence rates, and more successful vocational outcomes when treatment is concurrent (30, 33). Emphasis should also focus on why veterans are not being enrolled into VHA VR after a referral is made. After this identification is made, evidence-based interventions can be developed to (a) allow for vocational interventions to be implemented in MH or SUD treatment (prior to a referral is made), and (b) creating evidence-based interventions to increase program enrollment so that veterans obtain VR services that may lead to lower healthcare needs (e.g., fewer visits in VHA vocational rehabilitation, outpatient mental health, homelessness program, and medical hospitalizations) post-discharge (22, 25).

### Elimination of employment-related barriers

As evident by prior research, employment improves self-esteem, recovery, less reliance on medical and psychiatric services, and lesser symptom impact for people with mental illness (22, 25). Likewise, employment is a strong predictor of a higher quality of life, greater self-esteem, fewer psychiatric symptoms, and less absenteeism at work (34). Employment issues for people with medical and psychiatric disabilities/disorders are closely linked with an evolving body of legislation. Over time, laws are updated to keep pace with the changing nature of disability as medicine, science, societal attitudes, and education all advance. Of particular importance are the Rehabilitation Act of 1973, the Americans with Disabilities Act, and amendments for each. The Rehabilitation Act of 1973 is still significant today in that it challenged vocational rehabilitation programs to find new ways to support people with more severe impacts from disabilities to move into the workforce. This law also laid the groundwork for the Americans with Disabilities Act of 1990 (35) and the Americans with Disabilities Amendments Act [ADAAA] (36). The ADAAA affirmed and emphasized the right of workers and job seekers with disabilities to not only participate in the workplace, but also the right to access reasonable accommodations.

Reasonable accommodations are any changes to the workplace, job functions, or to the procedures surrounding the job that make it possible for the person with a disability to perform the essential functions of the job. It is important to note that the accommodation is meant to limit or eliminate the impact of the disability so that workers can perform their jobs. A few examples include changing the location of task-related supplies, modifying a work schedule: acquiring new equipment, or granting access to a private space for breaks. Reasonable accommodations are integral to promote the effectiveness of VR programs and have an impact on independent functioning, job satisfaction and retention, and quality of life indicators (37–39).

The Job Accommodation Network (JAN) provides a public resource for information about accommodations designed to support workers with disabilities and employers. Requests for accommodation ideas for individuals with mental health diagnoses are available. Some of the most common are flexible schedules, if there is required training this training may need to me modified, and a job coach (40). Furthermore, the least commonly requested accommodation type is a physical modification to a workplace for a person who has a mental health diagnosis. Although this is not a direct accommodation, several studies show that having a supervisor who is understanding and supportive lowers the risk factor for absenteeism and longer tenure for workers with mental health diagnosis (41). Potential accommodations for a person living with a mental health diagnosis such as anxiety, PTSD, Bipolar, or depression might include a flexible schedule, modified break schedules, a sound machine or quiet workplace, daily job tasks or itemized task lists, job restructuring, reminders, written instructions, noise canceling headsets, telework, and others as they may relate to supporting that individual.

There is significant psychological stress placed on an individual with severe mental illness and/or SUDs. Literature has found that it is hard for those diagnosed with MH disorders to request support or reasonable accommodations due to fear of retaliation and stigma (42–44). This shows the need for VHA VR services to assist in requesting workplace accommodations and helping the veteran manage work-related stress. The supported employment program within VHA VR uses the evidence-based Individual Placement and Support (IPS) model, which has shown high efficacy in employment outcomes (higher instances of employment and sustainment of employment) for those with serious mental illness due to the supports in requesting workplace accommodations and managing work-related stress (45). Eliminating these types of vocational barriers to employment can be vital is assisting the veteran to obtain and maintain employment despite significant employment related barriers (46).

### Study limitations

The current study had initial limitations that were addressed prior to analyzing the data. First, much of the data that were used in the current study was entered by VR counselors during the intake interview with the veteran. It is possible that this data were entered incorrectly into the database by VR counselors, therefore medical records in CPRS and JLV were used by the primary author to confirm that information was inputted correctly (the primary author made corrections if VR counselors inaccurately entered the information into the database – CPRS and JLV were deemed as more accurate than the database). However, it is still possible that data entry errors occurred, as information is manually inputted in CPRS and JLV systems. Another study limitation of the dataset was inaccuracy related to psychiatric diagnosis and past and current alcohol use and other substance use disorder. The database used during the intake interview did not account for the durational period of last SUD/AUD usage (active, early remission, sustained remission) nor psychiatric diagnoses per the DSM 5 TR (F-Code). Therefore, information was collected from CPRS and JLV to properly code the diagnoses (e.g., early remission from AUD or SUD is 3–12 months post last use). To account for stronger representation of access, enrollment, and efficacy of vocational rehabilitation services, a national study should be used to determine if the themes from the current study exist across the U.S.

## Conclusion

Prior research on VHA VR programs have primarily focused on outcomes. Current study findings have showed some disparities in program enrollment and employment at discharge when a veteran has a psychiatric diagnosis. With much focus of the VA related to access and outcomes, there is an apparent need for more research focus on how to increase program enrollment into VHA VR programs (why is the veteran not enrolling in services after referral is made).

## Data availability statement

The data analyzed in this study is subject to the following licenses/restrictions: The U.S. Department of Veteran Affairs has strict guidelines on the release of Protected Health Information. Due to the policies governing the release or availability of data, we are unable to release the data associated with the current manuscript. Requests to access these datasets should be directed to https://www.research.va.gov/programs/orppe/vacentralirb/default.cfm.

## Ethics statement

Ethical review and approval was not required for the study on human participants in accordance with the local legislation and institutional requirements. Written informed consent from the participants was not required to participate in this study in accordance with the national legislation and the institutional requirements.

## Author contributions

MS, FB, and HH contributed to the conception and design of the study. MS organized the database. MS completed the data analysis with the assistance of Y-SL. MS and Y-SL interpreted all data. HH, LR, FB, ZS, and AG wrote sections of the manuscript. All authors contributed to manuscript revision, read, and approved the submitted version.
